# Food Insecurity, Food Environments, and Disparities in Diet Quality and Obesity

**DOI:** 10.1093/geroni/igab046.1912

**Published:** 2021-12-17

**Authors:** Yeon Jin Choi

**Affiliations:** University of Southern California, Los Angeles, California, United States

## Abstract

Food insecurity is a public health concern that is associated with poor diet and obesity. Poor food environments with low access to healthy, affordable food may amplify the negative impact of food insecurity on diet and obesity. This study aims to investigate whether food insecurity and food environments are jointly associated with an increased risk of poor diet quality and obesity. We used data from a nationally representative sample of 6,395 older adults in the Health and Retirement Study Health Care and Nutrition Survey and the National Neighborhood Data Archive. Weighted regression models were estimated to examine the relationship between food insecurity and food environments with diet quality and obesity. Both food insecurity and poor food environment were associated with lower healthy eating index scores, indicating poorer quality diet. Food insecure older adults were more likely to be obese than food secure older adults and poor food environments exacerbate the negative impact of food insecurity on obesity risk. However, there was no statistical difference in obesity risk by food environment among food secure respondents. Findings from this study highlight the negative impact of limited access to healthy food due to financial difficulties and/or poor food environments on diet quality and obesity risk. Providing financial or nutritional supports along with efforts to promote healthy food environment may reduce disparities in diet quality and obesity. Special support should be provided to food insecure older adults with poor food environment, those at the greatest risk of poor diet quality and obesity.

